# Decentralized nursing education in Northern Norway: a basis for continuing education to meet competence needs in rural Arctic healthcare services

**DOI:** 10.3402/ijch.v73.25328

**Published:** 2014-09-12

**Authors:** Mari Wolff Skaalvik, Margrete Gaski, Bente Norbye

**Affiliations:** 1 Department of Health and Care Sciences, UiT The Arctic University of Norway, Tromsø, Norway; 2 National Centre of Rural Medicine, UiT The Arctic University of Norway, Tromsø, Norway

**Keywords:** RNs, continuing education, blended learning, rural Arctic region, professional competence, municipal health care service, recruitment, retention, off-campus

## Abstract

**Background:**

Ensuring a sufficient nursing workforce, with respect to both number and relevant professional competencies, is crucial in rural Arctic regions in Norway. This study examines the continuing education (CE) of nurses who graduated from a decentralized nursing programme between 1994 and 2011.

**Objective:**

This study aims to measure the extent to which the decentralized nursing education (DNE) in question has served as a basis for CE that is adapted to current and future community health care service needs in rural Arctic regions in northern Norway. More specifically, the study aims to investigate the frequency and scope of CE courses among the graduates of a DNE, the choice of study model and the degree of employment with respect to the relevant CE.

**Design:**

This study is a quantitative survey providing descriptive statistics.

**Results:**

The primary finding in this study is that 56% of the participants had engaged in CE and that they were employed in positions related to their education. The majority of students with decentralized bachelor's degrees engaged in CE that was part time and/or decentralized.

**Conclusions:**

More than half of the population in this study had completed CE despite no mandatory obligation in order to maintain licensure. Furthermore, 31% of the participants had completed more than one CE programme. The findings show that the participants preferred CE organized as part time and or decentralized studies.

Globally, there is a shortage of human health resources (1), and the need to recruit and retain health personnel in rural and remote areas has increased (1–5). Although rural areas contain approximately half of the global population, less than 38% of registered nurses (RNs) work in rural areas (1,6).

Northern Norway is facing specific challenges regarding health care service delivery (7). Approximately 470,000 people populate only one-third of Norway (7), and 56 of 88 municipalities have fewer than 3,000 inhabitants (7), yet most hospitals are located in a few urban centres. Further, statistical projections estimate that a shortage of 25,200 nurses will occur in Norway by 2035; thus, recruitment and retention of nurses remains a priority (7). While education, recruitment and retention of health professionals in general and nurses in particular are common concerns in rural settings internationally (8), to address this problem, a decentralized nursing education (DNE) has been implemented in northern Norway since 1990 (9). The DNE has been organized as a part-time, 4-year study in rural off-campus locations in order to offer a bachelor degree in nursing to candidates with responsibilities and obligations in their rural home communities. The curriculum has primarily been offered off-campus through established study groups supervised by university lecturers in the region. As communication technology became available, Internet-based communication as a learning management system, videoconferencing and on-line modules were included, combining the face-to-face learning in a blended learning approach (10). Supervised clinical placements were completed locally in nursing homes, home-based nursing and in psychiatric nursing facilities. Medical and surgical placements were conducted in an urban university hospital. This study model was initiated as a cooperation between the rural municipalities and the university, and has provided a stable nursing workforce for the rural health services in northern Norway (9).

Furthermore, the challenges of developing skilled and motivated health care personnel are critical to the provision of high-quality health services worldwide. The need for health services is complex because of the ageing population, which has complex health service needs that require advanced levels of care (1). Continuing education (CE) programmes for RNs can help ensure that RNs deliver high-quality health services and can assess whether RNs have updated knowledge and an in-depth understanding of nursing care.

This study presents an overview of the CE undertaken by nurses who graduated from the DNE in northern Norway between 1994 and 2012. It aimed to measure the extent to which the DNE has served as a basis for CE that is adapted to the current and future community health service needs. Specifically, this study investigates the frequency and scope of CE among graduates from the DNE, the choice of study model and the degree of employment in accordance with the applicable CE in rural Arctic areas in northern Norway.

## Health care reforms address the need for a variety of competencies

The Coordination Reform (CR) (11) implemented in Norway in 2012 addressed the need for expanded health service responsibilities at a municipal level. Core and expanded competencies are needed to improve outcomes for patients with chronic diseases, to increase patient safety and satisfaction and to shorten hospital stays (ibid). Necessary competencies are understood to be complementary types of knowledge that facilitate the provision of comprehensive health care services: *Competency is the habitual and judicious use of communication, knowledge, technical skills, clinical reasoning, emotions, values, and reflection in daily practice for the benefit of the individual and the community being served* (12). The guidelines in the CR (11) emphasize health service responsibilities with respect to geriatric care, palliative care and preventive health nursing in particular.

The CR was implemented because of the challenges of ensuring both equity and quality in health care services in a country characterized by a scattered population and 430 small municipalities (7). There are several challenges associated with the provision of health care services in rural Arctic settings, including challenges related to long travel distances, the Arctic environment, tourism and climate changes, which may involve a new and unknown disease panorama and acute conditions (13,14). To meet the intentions of the CR, the nursing workforce need to be sufficient, both in number and in relevant professional competencies.

## Nursing education and CE in Norway

The Norwegian nursing education is a 3-year bachelor (BA) programme covering 180 European Credit Transfer System (ECTS) points (15,16) provided by 33 universities throughout Norway. A national body (The Norwegian Registration Authority for Health Personnel), license RN's who have completed a BA in nursing. This allow RNs to work in all parts of the health services – healthcare institutions and in home-based nursing and in psychiatric care (16). A specific licence is required to work as a midwife after completion of midwifery education.

Once RNs are qualified with a BA in nursing, no CE is required to uphold the licensure. However, university hospitals require CE in anaesthetic nursing, operating theatre and intensive care nursing before employment in such units.

The term CE is frequently used synonymously with continuing professional development, continuing professional education, life-long learning and knowledge transition (17). In this paper, CE refers to continuatiion professional education that is part of the formal educational system in Norway acknowledged by ECTS points and offered by universities and university colleges in Norway (Table I). Until the 1980s, CE in Norway was limited with respect to both access to and choice of CE, similar to other European countries, such as Ireland (18). In addition to the programmes in the formal educational system, a number of courses in nursing topics are offered as clinical specializations and upgrading of competencies (19).

**Table I:** Continuing education programmes in Norway

Study programmes	Study points ECTS
Hospital specializations	90
Master's programmes	180
Geriatric nursing	90
Public health care	90
Midwifery	90
Nursing management	90
Mental health care	90
Clinical specializations	15–60

CE programmes for RNs are provided by universities and university colleges in Norway (20). Masters programmes have predominantly been offered as an academic career path; however, some universities now provide masters programmes in midwifery and mental health care, and an increasing number of clinical specialization programmes. This national trend has developed as a result of the Bologna Process (21). Clinical specialization programmes of shorter duration include palliative care, rehabilitation, diabetes and others.

In the Arctic region, CE programmes are generally offered as full-time programmes on campuses, yet certain programmes are provided on a part-time basis (19). Formal CE programmes cover 60–180 ECTS points. Because of the decentralized provision of health care services in Norway, there is a high need for specialist competencies throughout the country (11). An American Health Workforce analysis (8) reports that access to CE and professional development is necessary to maintain health workers’ competence and to improve their performance. CE programmes provided in rural areas aimed at the health care needs of rural and remote areas are likely to improve the competence of nurses working in rural areas (22,23). Nevertheless, RNs living and working in rural areas may have limited access to CE if it is only provided in full-time, on-campus programmes, since RNs seeking CE may have obligations that prevent them from being full-time students away from their residences (9).

## Method

### Design

This study is a quantitative survey providing descriptive statistics.

### Sample and participants

The participants in this study attended a DNE at The Arctic University of Norway (UiT), from 1990 to 2011. The graduated RNs responded to a questionnaire posted to their private addresses. From the sample of 356 former students, 41 questionnaires were returned because of unknown addresses. In total, 233 respondents returned questionnaires. Because of the missing participants, the findings cannot be considered representative of the entire population of graduated RNs who attended the DNE.

### The questionnaire

Because no validated questionnaires related to the study topic were found, the researchers developed a questionnaire (9). The questionnaire consists of background variables and 5 main subject areas with sub-questions addressing employment as an RN, rural or urban locations, work areas, CE and career paths. Responses were limited to the options presented in the questionnaire. The questionnaire was assessed by a panel of university lecturers before it was finalized, and a pilot study was conducted among 11 health care students to pre-test the questionnaire in 2011, leading to minor refinements. The survey was conducted in 2012.

### Data collection

The data were collected from RNs who had graduated from the DNE at the UiT. A research assistant recorded and coded the data from the questionnaires to ensure the anonymity of the respondents. An informational letter and a pre-paid return envelope were enclosed with the questionnaire. One reminder was posted to non-responders 2 weeks after the original reply date.

### Statistical analysis

The data were analyzed in SPSS 20.0 (www-01.ibm.com/software/analytics/spss/) with cross-sectional and descriptive univariate statistics for multiple variables. Summary statistics included frequency, mean, percent, and range for the cross-sectional and descriptive data (24).

### Ethics approval

The Norwegian Social Science Data Services (NSD) approved the study (approval number 27459), and the department head for the university's Department of Health and Care Sciences allowed the study to occur by releasing the names and addresses of the former students. The participants were then informed of the purpose of the study in an explanatory letter, and they were asked to participate in the study by completing and returning the provided questionnaire. The respondents’ informed consent was given by completing and returning the questionnaire in the attached envelope. Confidentiality and anonymity were guaranteed.

## Results

### Response rate

Of 315 RNs who graduated from the DNE between 1990 and 2011 and received questionnaires, 233 answered the questionnaire, for a response rate of 73.9%.

### Continuing education

Improved access to CE is important for nurses in rural areas because the cost of travel and time away from home increase the effort necessary to attend CE programmes (23), and professional development is necessary to maintain and increase competencies in health services. Among the respondents (i.e. RNs who graduated from the DNE [n=233] between 1995 and 2011), 56% have attended CE.

On a national level, 21% of the nurses have completed more than one CE course (19).

**Fig. 1:**
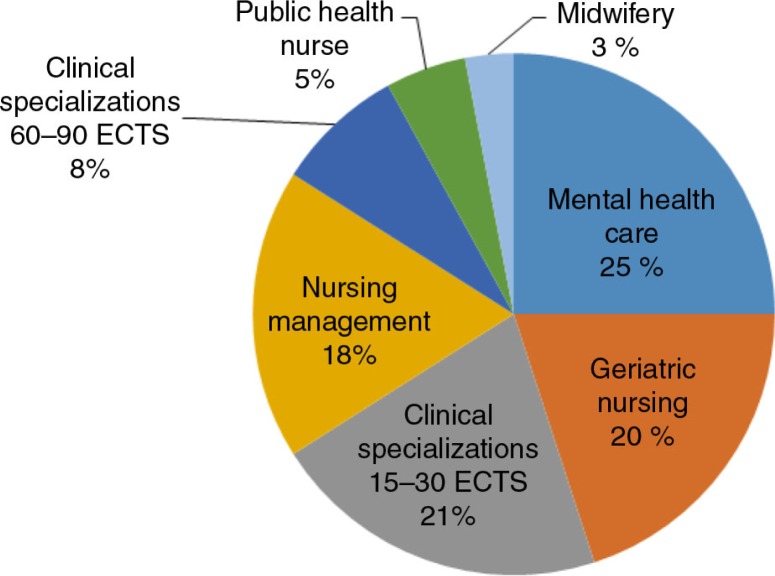
Categories of continuing education among RNs educated via decentralized nursing education.

The participants predominantly attended CE programmes closely related to municipal health care responsibilities, such as geriatric and mental health care. Public health care and midwifery services are also important tasks in municipal health services, but fewer positions are available in these areas relative to elderly and mental health care. To have a hospital specialization, RNs must have a clinical specialization of 60–90 ECTS points in areas such as intensive care nursing, anaesthetic nursing, paediatric care and operating room nursing. Clinical specializations of shorter duration cover 15–30 ECTS points. Four of the respondents held master's degrees in subjects related to nursing management and clinical specializations. The categories of CE are presented in Figure 1 according to the specialization.

### Study model

Part-time and/or decentralized models of CE programmes are preferred among the respondents holding a bachelor's degree in nursing from a decentralized education model in northern Norway.

### Leadership in rural health care

In municipal health services, RNs constitute the largest proportion (1) of health professionals, yet well-organized health care service requires good leadership (25). RNs can receive CE in administration and management through several educational paths (20). In this study, competence in leadership varied among participants holding a management position.

## Discussion

CE is available to a large proportion of the nursing workforce in Norway, as most of the programmes (Table I) are delivered in each of the 19 counties in Norway, including Troms, where the participants in this study live and work (9). In this study, the importance of access to CE is viewed from a broader perspective than mere knowledge acquisition. Rural Arctic municipalities generally have small organizational health care units and require a wide range of tasks and responsibilities that pose challenges with regard to the necessary competencies for the provision of health care services. Attending CE programmes provides RNs with opportunities to interact with other RNs and to maintain professional networks (1), which promotes the retention of health personnel in rural Arctic areas and increases the diversification of health care services provided. Furthermore, professional networks across municipalities can expand inter-municipal cooperation and can facilitate expertise and service sharing.

According to the CR (11), local services must be developed to prevent unnecessary hospitalizations. Further, long travel times to hospitals should be avoided for fragile patients who can be cared for in their local communities, particularly in Arctic environments (14). Thus, CE is required to facilitate health professional's acquisition of necessary competencies. Health care reforms in the 21st century call for increased access to CE in line with future service needs (26).

In this survey, 56% of the respondents had completed CE (Table II). Thus, they received the necessary specialization to work in a rural Arctic environment that is characterized by a wide range of professional tasks requiring enhanced knowledge and competencies. Because of non-responding and missing respondents, the findings may not be representative of Norway in general; however, the results correspond with findings reported in a study from 2009 (19). Elderly care is a major task in municipal health services; such care is often provided in nursing homes and at patients’ homes, and RNs require competence in geriatric nursing to provide elderly care. Competence in geriatric nursing is especially important because the prevalence of dementia is predicted to increase (27).

**Table II:** Frequency of continuing education among RNs from a Norwegian decentralized nursing programme (N = 233)

	Female		Male		Total	
	
	Frequency	%	Frequency	%	Frequency	%
Continuing education	126	58	5	33	131	56
No continuing education	88	40	10	67	98	42
Missing	4	2	0	0	4	2
Total	218	100	15	100	233	100

Increasing life expectancy in conjunction with chronic conditions will also increase the need for competence in palliative care. Philips et al. (28) reported that CE programmes that are designed for rural nurses should be multifaceted and should address participants’ needs and learning preferences to improve end of life care. Barriers such as costs and access affect the possibilities for undertaking CE for professionals living in rural areas (28).

To achieve the aims of the Coordination Reform, RNs will need to attend CE as a part of life-long learning (11) to ensure that the quality and equity of health service provision at a municipal level is comparable to that in urban areas. Beatty (23) found that 86% of the studied sample had participated in CE in the past 2 years; further, more than 60% of the respondents reported participating in 3 or more CE activities, and 21% reported participating in more than 7 CE activities. In the present study, 7% of the respondents had completed more than 2 CE programmes.

In rural Arctic regions of Norway, municipal health services are the primary providers of services to the population (27). Before the implementation of the CR (11), CE in geriatric, mental and public health care was beneficial from an employee's perspective because it increased their employability. Changes in how health care services are organized owing to the transfer of tasks from specialist health services to the municipal health service level (11) will alter the need for competencies in rural Arctic areas and will likely influence professionals’ motivation for and choice of CE programmes in the future. CE in mental health care and geriatric nursing was the most common CE received by the participants in this study, and these competencies coincide with the existing and future needs for competencies in municipal health care services. CE in midwifery and public health nurse education was received by 8% of the participants (Table III). Because of Norwegian health care service reforms, there is an ongoing debate regarding the provision of midwifery services in rural areas, especially concerning maternity units (29). An eventual shift in the provision of midwifery services may reduce the number of positions for midwives in rural areas, even though prenatal care is a statutory task (29). Preventive health care is a priority according to Norwegian health policy (11), and this focus on preventive health care has resulted in increased financial transfers to municipalities.

**Table III:** Distribution of the participants with respect to study organization (N=131)

	Frequency	%
Part time	62	47
Decentralized	29	22
Full time	19	15
Part time and full time	7	5
Part time and decentralized	3	2
Other	1	1
Missing	10	8
Total	131	100

In total, 186 CE programmes were completed by the RNs in this study (n = 131). Furthermore, 41 of the respondents completed more than 1 CE, and 17 completed 3 different CE courses. These results show the need for competencies to safeguard and facilitate the execution of complex and varied tasks in municipal health services. The findings indicate that the participants experienced a need for more than 1 specialization in a health service that is characterized by diverse and complex tasks and responsibilities. The findings can also be understood to indicate the ongoing need to upgrade skills and competencies from a life-long learning perspective (30). Based on the findings, the participants who had participated in CE held working positions in line with their achieved qualifications (9). This finding is somewhat surprising, as many of the respondents live in small municipalities (e.g. less than 3,000 inhabitants) with correspondingly limited work opportunities. The employment rate for midwives, for example, may reflect the fact that some RNs are employed in municipalities near the municipality where they lived.

Since the 1990s, delivery of study programmes has been characterized by policy guidelines emphasizing distributed education and blended learning (10). The participants in this study had experiences from a decentralized study model in which the majority of the education occurred in rural Arctic municipalities (9). This study model places demands on students in terms of motivation and individual responsibility for the learning process and presents challenges for students to combine their working life with their education. Therefore, this study examined how experiences from being a decentralized nursing student influenced the students’ choice of study model for CE. Among the population of nurses attending CE (n = 131), the majority (Table IV) attended part-time and/or decentralized programmes. Thus, the respondents’ experiences from their bachelor's education did not deter them from choosing this study model for CE.

**Table IV:** Distribution of continuing education among participants in management positions (N = 47)

Continuing education	Frequency	%
Continuing education, clinical disciplines	24	51
Continuing education, management	10	21
Unspecified continuing education	1	2
No continuing education	12	26
Total	47	100

Smyth et al. (31) found that a blended learning postgraduate programme has the potential to contribute to practice and to enhance learning. Black and Bonner (32) stated that distance education is the vehicle for the delivery of CE. The findings of the study showed that 28% of the participants enrolled in further courses via distance education and that 54% of the participants planned future studies. The respondents reported a strong desire for support in obtaining CE from their employers. Employer support for CE was not examined in the present study, but because distance, time, and work obligations limited RNs’ ability to attend CE, employer support and facilitation is likely important.

Health care services are rapidly changing (11,26,33). Plans and strategies for future municipal health service needs require qualified leaders who can foresee health care challenges and who can plan for the recruitment of health personnel with necessary competencies. Nurses entering leadership positions should have competence and insights regarding the required competencies so that they can promote opportunities to combine work and education for employees of rural Arctic health services. Based on the findings in this study, less than one-fourth of the participants in nursing management positions had received CE in this field. CE in management is thus an area that needs to be strengthened (11,34).

### Limitations

The results of this study are limited because of the lack of data from RNs who did not receive the questionnaire and non-respondents. The small sample and the fact that no similar studies were found for comparisons imply that the findings cannot be generalized.

### Implications for practice

In a society with a rapidly changing health care system, policy makers and nurse educators must evaluate and revise their approaches and programmes to ensure that current and future needs are met. Multifaceted and interdisciplinary CE programmes based on health care needs in rural Arctic areas should thus be developed. Close cooperation between educational institutions and health services should be established to secure that CE programmes match the needed competencies for rural Arctic health services. Furthermore, regulatory, financial, personnel and professional support interventions are vital factors to promote and sustain an appropriate workforce in rural Arctic areas. Research among RNs working in rural areas is recommended in order to obtain knowledge about factors that promote and limit conducting CE. This is pivotal in order to contribute to equity in rural Arctic and urban health services with respect to the special challenges in the Arctic regarding recruitment, retention and relevant professional knowledge and competencies.

## Conclusions

The results of this study show that more than half of the participants were motivated for CE. Regarding future needs for competencies in rural Arctic health services, this relatively high number needs to increase. The fact that 31% of the participants attended more than 1 CE is understood as a consequence of the wide range of tasks and responsibilities for RNs in rural Arctic areas. Furthermore, the findings indicate that the participants’ previous experiences with a decentralized, flexible study model were positive and that they preferred CE organized as part time and/or as decentralized studies.
